# Evaluation of a standard provision versus an autonomy promotive exercise referral programme: rationale and study design

**DOI:** 10.1186/1471-2458-9-176

**Published:** 2009-06-08

**Authors:** Kate Jolly, Joan L Duda, Amanda Daley, Frank F Eves, Nanette Mutrie, Nikos Ntoumanis, Peter C Rouse, Rekha Lodhia, Geoffrey C Williams

**Affiliations:** 1 School of Health & Population Sciences, University of Birmingham, Birmingham B15 2TT, UK; 2 School of Sport and Exercise Sciences, University of Birmingham, Birmingham B15 2TT, UK; 3 Department of Sport, Culture and the Arts, Strathclyde University, Glasgow G13 1PP, UK; 4 Department of Clinical and Social Sciences in Psychology, University of Rochester, Rochester, New York 14627, USA

## Abstract

**Background:**

The National Institute of Clinical Excellence in the UK has recommended that the effectiveness of ongoing exercise referral schemes to promote physical activity should be examined in research trials. Recent empirical evidence in health care and physical activity promotion contexts provides a foundation for testing the utility of a Self Determination Theory (SDT)-based exercise referral consultation.

**Methods/Design:**

Design: An exploratory cluster randomised controlled trial comparing standard provision exercise on prescription with a Self Determination Theory-based (SDT) exercise on prescription intervention.

Participants: 347 people referred to the Birmingham Exercise on Prescription scheme between November 2007 and July 2008. The 13 exercise on prescription sites in Birmingham were randomised to current practice (n = 7) or to the SDT-based intervention (n = 6).

Outcomes measured at 3 and 6-months: Minutes of moderate or vigorous physical activity per week assessed using the 7-day Physical Activity Recall; physical health: blood pressure and weight; health status measured using the Dartmouth CO-OP charts; anxiety and depression measured by the Hospital Anxiety and Depression Scale and vitality measured by the subjective vitality score; motivation and processes of change: perceptions of autonomy support from the advisor, satisfaction of the needs for competence, autonomy, and relatedness via physical activity, and motivational regulations for exercise.

**Discussion:**

This trial will determine whether an exercise referral programme based on Self Determination Theory increases physical activity and other health outcomes compared to a standard programme and will test the underlying SDT-based process model (perceived autonomy support, need satisfaction, motivation regulations, outcomes) via structural equation modelling.

**Trial registration:**

The trial is registered as Current Controlled trials ISRCTN07682833.

## Background

Within the media, public health sector, and policy making communities, attention has been increasingly drawn to the obesity epidemic and corresponding sedentary lifestyles that are escalating in society. The promotion of physical activity, of a sufficient intensity, duration, and frequency, has been identified as a means to counteracting these worrying trends. Moreover, levels of physical activity have been linked to a range of health-related outcomes including all-cause mortality [1] and coronary heart disease (CHD) and hypertension [2,3], some cancers [4], and the aetiology of type II diabetes mellitus [5,6]. Regular physical activity is also implicated in the maintenance of functional capacity, muscular skeletal health [7] and psychological well-being [8-10].

Although recent US government guidelines have distinguished between the minutes per week required as a function of whether the physical activity is moderate or of high intensity [11], Haskell and colleagues have recommended the minimum frequency and duration of moderate to vigorous physical activity necessary to gain such health benefits. These recommendations, which have been endorsed by the UK government [12] and the American College of Sports Medicine, state that to promote and maintain health, adults aged 18–65 years need to engage in moderate-intensity aerobic (endurance) physical activity for a minimum of 30 minutes (in durations of at least 10 minutes) on at least five-days each week or vigorous-intensity aerobic physical activity for a minimum of 20 min on three days each week. A large percentage of the UK population, however, do not meet these current active living recommendations and thus are less likely to accrue health benefits that an active lifestyle might provide [13]. In an attempt to increase physical activity levels in the general population, a range of interventions have been developed and implemented and systematic review suggests that interventions that are theory driven and use a counselling style to change behaviour have evidence of successful behaviour change [14].

In recent years, there has been a focus on the role that primary care should play in identifying and promoting physical activity as a behaviour relevant to the adoption and continuance of a healthy lifestyle [15]. In the UK, Primary Care Trusts and local authorities have implemented exercise referral schemes that make initial use of the personal relationship that exists between a general practitioner (GP) and patient. Generally, these schemes commence with a GP or practice nurse referral of an individual deemed to possess at least one major risk factor for cardiovascular disease to a health and fitness advisor (HFA) located at a community leisure centre.

Evidence investigating the effectiveness of exercise referral programmes is limited and the research that does exist provides mixed evidence [16]. Dugdil et al. [17] conducted a critical review of the development, impact and evaluation of exercise referral schemes and found that adherence rates were 35–45% and, although physiological changes were statistically significant, the magnitude of change was not sufficient to convey any health benefits. Even less is known about the impact of such exercise referral schemes on the long-term physical activity engagement and mental health of individuals who have participated in such programmes. A recent systematic review has evaluated and synthesised the evidence on the effectiveness of primary care initiated exercise referral in terms of changes in physical activity as well as physical and psychological health outcomes [16]. This review identified six randomised controlled trials (RCTs) comparing an exercise referral scheme with usual care as well as using the findings from other study designs to estimate drop-out rates from these schemes. All took place in the UK and five were individually randomised and one was a cluster RCT. A meta-analysis was undertaken using the proportion of the participants who were moderately active at follow-up (defined as taking at least 90–150 minutes of moderately intense activity per week). The relative probability of being moderately active was significantly higher among participants in the exercise-referral schemes (RR 1.2, 95% CI 1.06 to 1.35) compared to usual care. Other outcomes, such as body mass index (BMI), waist-hip ratio, percentage body fat, resting heart rate, blood pressure and cholesterol level were measured in three of the trials. Whilst these outcomes improved in the exercise referral arms, they also improved in the control groups resulting in no statistically significant differences between the groups. One trial measured psychological morbidity [18]. Whilst anxiety, as measured by the Hospital Anxiety and Depression scale (HADS), improved in exercise and control groups to 6-months follow-up, only the participants in the exercise group exhibited a significant improvement in HADS depression score.

Following a recent appraisal of the evidence on the effectiveness of exercise referral schemes for the National Institute for Health and Clinical Excellence (NICE; [19] p 23), it was concluded that: "Exercise referral schemes can have positive effects in the short-term (6 to 12 weeks), but are ineffective in increasing activity levels in the longer term or over a very long time frame (over 1 year)." As a result, NICE [19] (p 6) recommended that "practitioners, policy makers, and commissioners should only endorse exercise referral schemes to promote physical activity that are part of a properly designed and controlled research study to determine effectiveness." The present trial is a response to this guidance.

### Evaluation of an Exercise Referral Programme

Variations between exercise referral models exist across the UK. Therefore a thorough description of the targeted programme is essential. The present trial entails an evaluation of a service that is available to all Birmingham residents and or patients registered with a Birmingham PCT GP (Birmingham East & North PCT; South Birmingham PCT and Heart of Birmingham teaching PCT). The 'Exercise on Prescription' (EOP) scheme aims to facilitate the adoption and maintenance of increased physical activity levels by sedentary patients identified in Primary Care, with the underlying aim of improving their physical and mental health.

Entry into the EOP scheme typically commences (i.e. some patients are self referred) by either a GP or Practice Nurse identifying a patient who would benefit from being more physically active and is judged to have the motivation to increase his/her physical activity levels. The GP or practice nurse completes a prescription card that acts as the referral documentation and clearly states the relevant information about the patient's health status. The health and fitness advisors (HFAs), who are exercise professionals working to Level three of the National Occupational Standards, subsequently arrange an initial 1 hour consultation at a local leisure centre. During this consultation, the HFA asks the client about their current state of health, medical problems, medications taken and current activity taken then the HFA and client negotiate and agree an appropriate programme of individual and/or group activities which will help the patient to achieve the desired outcomes. In the following 10–12 weeks, the client takes part in the exercise programme with support provided by the HFA as required. At the conclusion of the programme, the HFA invites the patient to an exit consultation to discuss future participation in physical activity.

Consistent with the recommendations of the recent NICE guidance on methods to increase physical activity [19], one aim of the trial is to determine the effect of the standard provision exercise referral scheme operating in Birmingham on participants' self-reported physical activity, associated health behaviours, physical health, and well-being/quality of life at 3 months and at a 6 month follow-up.

### Self Determination Theory-based Exercise Consultation

The literature centred on physical activity promotion has advocated the relevance of theory to the design, delivery and evaluation of exercise referral schemes [14]. In line with this consideration, the present trial also entails the development and preliminary testing of a training programme for health/exercise advisors grounded in Self Determination Theory [20,21]. SDT is a contemporary theory of motivation centred on the social psychological processes underlying variability in behavioural adoption, maintenance, and optimal functioning/well-being. SDT is concerned with the "why" of behavioural regulation and, in particular, centres on the degree to which people's motivation toward behavioural engagement and behaviour change (such as increasing levels of physical activity) is self-determined or controlled by external factors or internalised contingencies (such as guilt). According to SDT, all individuals have the need to feel competent, autonomous, and connected with others (i.e., experience relatedness). If these needs are met, self determined motivation should be promoted and well-being enhanced. SDT assumes people are intrinsically oriented toward growth and health, and will naturally internalise self-regulations regarding behaviours. Internalisation is enhanced if the psychological needs are supported and SDT also proposes that autonomy-supportive interactions with significant others (such as HFAs) contribute to satisfaction of the three psychological needs of competence, autonomy, and relatedness and more self-determined reasons for doing an activity. Within autonomy supportive exercise consultations, HFAs would offer choice in activities, acknowledge participants' perspectives, minimise external rewards and demands regarding becoming more active, provide meaningful rationales for physical activity engagement, and support personal choice regarding initiations to change.

Evidence is growing regarding the implications of self determination for health behaviours and enhanced well-being. For example, SDT-based research has shown that more self-determined regulations can predict adherence to medical prescriptions [22], smoking cessation [23,24], weight loss [25], glycemic control [26], physical activity engagement [27-31], and adherence to exercise referral schemes [31]. Specific to participation in physical activity, previous studies have supported the hypothesised links between need satisfaction, autonomous motivation and indicators of more positive mental and emotional health. Further, past work has provided evidence for the positive role of autonomy supportive consultations on behavioural change [23,30]. Thus, a strong theoretical and empirical foundation exists for testing the utility of an SDT-based exercise intervention in the context of exercise referral.

**Figure 1:**
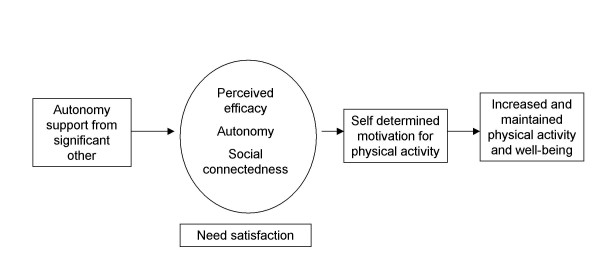
**The main tenets of self-determination theory to be examined, tested and explored**.

As a result, the present trial compares the effect (at 3 and 6 months) of an exercise consultation delivered by SDT-trained HFAs with a standard exercise consultation provided by trained HFAs in Birmingham on participants' self-reported physical activity, associated health behaviours, physical health, and well-being/quality of life. Our prior hypothesis was that participants in the SDT-arm would have more sustained physical activity and thus would report more activity at the 6-month follow-up. The conceptual model underlying the intervention is presented in Figure 1.

## Methods/Design

An exploratory cluster randomised controlled trial with an SDT-based intervention and standard provision exercise referral comparison arm was conducted. Ethical approval was obtained from the School of Sport and Exercise Sciences Ethical Review Committee at the University of Birmingham. The 13 leisure centres providing exercise referral in Birmingham were randomised to current standard practice or to the intervention arm; the HFAs working at the centres randomised to the intervention arm received self determination theory training. Randomisation was stratified by Primary Care Trust (PCT) and deprivation of population served and undertaken by an independent statistician.

The study commenced with a pilot phase in which the intervention (including the training of the HFAs, adequacy of the assessment tools for the targeted population, and preparation of the self determination theory-based booklets) were evaluated qualitatively via one-on-one interviews. Throughout the trial, the adequacy of the consultation style of the self determination theory trained HFAs was examined via videotaping/recording of a random sub-sample of participant face-to-face consultations. Specifically, to further examine the extent to which the intervention is implemented with fidelity to the theoretically-based protocols [32], an observational instrument was developed that assesses the interpersonal style and autonomy supportive behaviours and strategies employed by HFAs during physical activity consultations [33].

Differences in the perceived autonomy-supportive features of the exercise consultation among participants in the intervention, in contrast to the standard exercise on prescription provision, are also examined.

Participants in this trial are those people referred to the exercise referral scheme by their GP or practice nurse, who agreed to be part of the study (over a 7 month recruitment period). Each participant received the intervention consistent with their assigned HFA. Consent to follow-up as part of the study was taken by the HFA and the assessments of primary and secondary outcomes taken at baseline, three and six months.

### SDT-based intervention

The intervention spans a three month period during which an HFA aims to have one-to-one contact, in person (at leisure centres) or via telephone, with participants on four occasions. This parallels the autonomy-supportive protocol employed by Williams et al. [34] in their RCT focused on smoking cessation/dietary change and also is in line with the findings of Hillsdon et al.'s [35] review regarding the recommended frequency of intervention occasions. The one-on-one interactions also aimed to be consistent with guidelines for conducting exercise consultations in the literature [36] and aligned with non SDT-based research concerning effective health care provider-client interactions [37].

Following the baseline assessment of the primary and secondary outcomes, the initial consultation comprised a 1 hr one-to-one person centred interview. The initial consultation focused on a discussion of the benefits and risks of increased physical activity (individualised to the client's views about the consequences of regular physical activity and personal health risk(s)). Each client has the opportunity to discuss their exercise history, views regarding the advantages and disadvantages of change regarding physical activity level, the perceived barriers to and resources for change, views regarding how their intention(s) to become more active might be implemented, and the current and potential offerings of social support regarding exercise engagement. Goal setting takes place with the discussion centred on what specific activities the client intends to engage in, for how long, and when [in terms of day(s) and time(s)] in the upcoming week.

At the conclusion of the consultation, participants had the option of taking part in a fitness appraisal (consistent with the standard exercise referral scheme) and were given a self-management exercise promotion booklet designed to encourage a more autonomous perspective on physical activity initiation ("Empowering your Life with Exercise"). This booklet was developed from existing and successful physical activity promotion materials in the literature (e.g. the "Walk in to Work Out" pack [38] and the Diabetes Prevention Program's Lifestyle Change Program Manual [39] but consonant with the constructs and tenets of self determination theory.

At 1 month, the next contact (15–20 min) is conducted via telephone or in person. This consultation centres on: supporting all attempts the client made to be more active even if he/she failed at sustaining behavioural change, normalizing failure as expected when people are trying to change their behaviour, problem solving with the client to enhance and maintain his/her self efficacy or confidence regarding physical activity engagement and circumvent barriers to regular participation that have emerged, and recalibrating goals to align with client's willingness to become more active and confident in his/her ability to be more active.

At 2 months, a brief (5 min) phone call or in person interaction by the advisor is made to offer encouragement regarding all or any attempts by the client to be physically active, brainstorm with the client about how to address any new or retained barriers to physical activity, and discuss the client's goals for the next several weeks of the programme.

At 3 months, primary and secondary outcomes are re-assessed and a final face-to-face or via telephone "booster" consultation (20–30 min) takes place focused on recognising and reinforcing the internalisation of the participant's physical activity involvement, feelings regarding engaging in physical activity and being more active, and planning for future maintenance of physical activity. Again, the option of a fitness appraisal is made available. A supplemental self-management booklet centred on the monitoring and maintenance of physical activity is provided at this time.

### Training

**Figure 2:**
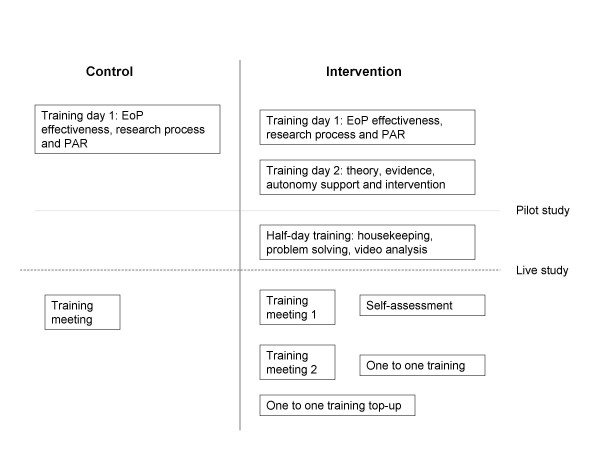
**The training protocol received by the control and intervention arms**.

Few studies have reported the protocol by which a professional may be trained to implement an intervention. It is critical that protocols are standardised, and reported in detail so that successful interventions may be replicated [40]. Figure 2 provides a summary of the training that the standard provision and SDT-based arms received. All training was undertaken by members of the research team.

#### Training of 'standard provision' HFAs

Those HFAs, randomly assigned to the standard provision arm, received and attended Training Day One. This training involved presentations that summarised the research pertaining to the effectiveness of exercise referral and the importance of evaluation (as defined by the NICE guidelines) [19]. The research process that was being undertaken was discussed and an explanation and demonstration of the 7 day Physical Activity Recall was conducted. Following the commencement of the study, a further training meeting was held between two research team members and the usual EoP arm HFAs which focussed on issues of recruitment.

#### Training of SDT-based intervention HFAs

The HFAs, who were randomly assigned to the intervention arm, attended the same training day as the standard provision arm but this was supplemented by a second training day. Training Day Two explained the theoretical background (SDT) of the intervention, explored the principles of client-centred verbal (e.g., expressing empathy, listening and parroting) as well as non-verbal (e.g., making eye contact) communication [41], discussed how to create an autonomy-supportive environment and reviewed strategies to promote the client's autonomous motivation for behavioural change. The HFAs were given the opportunity to conduct role plays implementing the autonomy supportive strategies and allowing practice of the skills relevant to effective communication.

The SDT-based intervention HFAs received a second phase of training having conducted the pilot study for 2 months. A half day training session focussed on the implementation of the self determination theory based intervention by discussing, viewing and analysing example video consultations, and problem solving any issues that had surfaced. After the conclusion of this training session, the HFAs indicated their satisfaction with the training that they had received and were confident in their ability to start live data collection. However, training remained integral throughout the trial and was a continuous process.

Once live data collection commenced, the first "top-up" session was held between a research team member and the SDT-based intervention arm HFAs. A self assessment sheet, a self reflective DVD (containing a videotaped file of each respective HFA conducting a live consultation) and an autonomy supportive rating sheet were provided for each HFA as a method of self-appraisal and self-evaluation.

Approximately two months into the trial, a third phase of training was provided for the SDT-based intervention arm. This third training session comprised a group discussion centred on addressing any questions or problems that the HFAs were experiencing. This was complemented by an on-site, one to one training session, referencing recorded videotaped clips of the respective HFA engaged in a recent consultation.

A final phase of one to one on-site training took place in the fourth month of the live trial. This training aimed to act as a refreshment of what had been taught previously and troubleshooting any issues that may have arisen.

### Outcome measures

The primary outcome is self-reported physical activity using the 7-Day Physical Activity Recall [42] assessed via telephone to maintain blinding. Estimated overall energy expenditure and time spent in moderate to vigorous intensity physical activity will be calculated for all participants at 3 time points (baseline, 3 months and 6 months).

Secondary outcomes included (1) physical health outcomes – BMI, BP (both measured at baseline and 6 months only), health status (Dartmouth CO-OP Charts) [43,44], (2) Mental/emotional well-being-anxiety and depression measured by the Hospital Anxiety and Depression Scale [45], vitality using the Subjective Vitality Scale [46,47], and other scales embedded in the Dartmouth CO-OP Charts), and (3) Motivation and processes of change – perceptions of autonomy support from the advisor (i.e., the Health Care Climate Questionnaire [25], perceived competence, autonomy, social connectedness or relatedness with respect to physical activity (via the Psychological Need Satisfaction in Exercise Scale) [48], and motivational regulations for exercise (via the BREQ-2 [49]).

### Follow-up assessments

At 3-months, the PAR was administered over the telephone by trained members of the research team who were blind to the participants' group allocation. Questionnaires were posted to participants as it emerged that few 10–12 week assessments by the HFAs took place in person. Questionnaires were administered over the phone to non-responders by post.

At 6 months, participants were requested to come to a leisure centre for their BP and BMI assessments. At that time, the PAR was administered and other secondary outcomes assessed via questionnaire. Those who did not attend the leisure centres were posted the questionnaires and asked to do the PAR over the telephone.

### Numbers of recruits and power of the evaluation

A sample size of 494 participants (38 each from 13 clusters) would provide a sample large enough to detect a difference in mean physical activity time across the 2 groups of 100 minutes with 80% power and 5% significance level. This estimate is based on a standard deviation of 211 mins (Jakicic et al) [50] and intracluster correlation coefficient of 0.04 (Eldridge et al) [51]. This sample size is sufficient to achieve 90% power and 5% significance to detect a within group increase of 60 minutes of self-reported physical activity from 108 (sd 211) at baseline.

### Analytical Strategy

Differences in primary and secondary outcomes between control and intervention groups will be compared using intention to treat analysis. At each stated follow up point, outcomes will be compared using multivariate regression based methods (e.g. logistic or least squares) adjusting follow up scores for baseline scores (where available) and key baseline characteristics (e.g. age/sex, race/ethnicity). Subgroup effects will be explored using interaction tests. Given that the study is likely to be inadequately powered for such subgroups, these analyses will be exploratory rather than inferential. Imputation methods will be used to assess data losses through level drop out and loss to follow up. All results will be reported as means and 95% confidence intervals. Similar to studies by Williams and colleagues [24,26], we will also test via structural equation modeling a theory-based process model to examine whether SDT-based personal and situational variables can predict changes in physical activity and well-being over time.

## Discussion

In this article, we have set out the rationale for further exploration of how to promote physical activity through exercise referral schemes. We have also outlined the key components of an explorative RCT that evaluates a standard provision exercise referral scheme while developing, implementing and comparing a parallel intervention grounded in SDT [20,21]. SDT offers significant insights into the processes by which different health behaviours may be changed and maintained [24,26]. Evidence suggests that this theory deserves application and evaluation with respect to the adoption and maintenance of physical activity [29,31].

If we are to continue to make progress in our understanding of how we can facilitate health behaviour changes, it is critical that future research groups report, in published sources, systematic and detailed descriptions of their study designs, training programmes and intervention protocols. Only when intervention content is documented and the method of evaluation is transparent will there be greater knowledge of how we can foster behavioural change aimed at improving quality of life.

## List of abbreviations

BMI: body mass index; BP: blood pressure; EoP: exercise on prescription; GP: general practitioner; HADS: Hospital Anxiety and Depression Scale; HFA: health and fitness advisor; NICE: National Institute for Health and Clinical Effectiveness; PA: physical activity; PAR: physical activity recall; SDT: Self Determination Theory; UK: United Kingdom.

## Competing interests

The authors declare that they have no competing interests.

## Authors' contributions

KJ, JD, AD, FE, NM, NN and GW designed the study and wrote the initial protocol. KJ, JD, FE, NM, RL, PR and GW delivered elements of the training programme. RL and PR co-ordinated the study with supervision from KJ and JD. KJ, JD and PR drafted the manuscript. All authors read and approved the final manuscript.

## Pre-publication history

The pre-publication history for this paper can be accessed here:



## References

[B1] Lee IM, Skerrett PJ (2001). Physical activity and all-cause mortality: what is the dose-response relation?. Med Sci Sports Exerc.

[B2] Talbot LA, Morrell CH, Metter EJ, Fleg JL (2002). Comparison of cardiorespiratory fitness versus leisure time physical activity as predictors of coronary events in men aged < = 65 years and >65 years. Am J Cardiol.

[B3] Jolliffe JA, Rees K, Taylor RS, Thompson D, Oldridge N, Ebrahim S (2003). Exercise-based rehabilitation for coronary heart disease.

[B4] Thune I, Furberg A-S (2001). Physical activity and cancer risk: dose response and cancer, all sites and site-specific. Med Sci Sports Exerc.

[B5] Helmrich SP, Ragland DR, Leung R, Paffenbarger RS (1991). Physical activity and reduced incidence of non-insulin-dependent diabetes mellitus. New Eng J.

[B6] Diabetes Prevention Program Research Group (1991). Reduction in the incidence of type 2 diabetes with lifestyle intervention or metformin. New Eng J Med.

[B7] Vuori M (2001). Dose-response of physical activity and low back pain, osteoarthritis, and osteoporosis. Med Sci Sports Exerc.

[B8] Lawlor DA, Hopker SW (2001). The effectiveness of exercise as an intervention in the management of depression: Systematic review and meta-regression analysis of randomized controlled trials. BMJ.

[B9] Sjosten N, Kivela SL (2006). The effects of physical exercise on depressive symptoms among the aged: A systematic review. Int J Geriatr Psychiatry.

[B10] Dunn AL, Trivedi H, Kampert JB, Clark CG (2005). Exercise treatment for depression: efficacy and dose response. Am J Prev Med.

[B11] US Department of Health & Human Services (2008). Physical activity guidelines for Americans.

[B12] Haskell WL, Lee IM, Pate RR, Powell KE, Blair SN, Franklin BA, Macera CA, Heath GW, Thompson PD, Bauman A (2007). Physical Activity and Public Health: Updated Recommendation for Adults from the American College of Sports Medicine and the American Heart Association. Med Sci Sports Exerc.

[B13] Department of Health (2004). At least five a week: Evidence on the impact of physical activity and its relationship to health.

[B14] Kahn EB, Ramsey LT, Brownson RC, Heath GW, Howzw EH, Powell KE, Stone EJ, Rajab MW, Corso P (2002). The effectiveness of interventions to increase physical activity – A systematic review. Am J Prev Med.

[B15] Fortier MS, Tulloch H, Hogg W (2006). A Good Fit: Integrating physical activity counselors into family practice. Contemp Educ Psychol.

[B16] Williams NH, Hendry M, France B, Lewis R, Wilkinson C (2007). Effectiveness of exercise-referral schemes to promote physical activity in adults. Br J Gen Pract.

[B17] Dugdill L, Graham RC, McNair F (2005). Exercise referral: the public health panacea for physical activity promotion? A critical perspective of exercise referral schemes; their development and evaluation. Ergonomics.

[B18] Issacs AJ, Critchley JA, See TaiS, Buckingham K, Westley D, Harridge SDR, Smith C, Gottlieb JM (2007). Exercise Evaluation Randomised Trial (EXERT): a randomised controlled trial comparing GP referral for leisure centre-based exercise, community-based waking and advice only. Health Technol Assess.

[B19] National Institute for Health & Clinical Excellence (2006). Four commonly used methods to increase physcial activity: Brief interventions in primary care, exercise referral schemes, pedometers and community-based exercise programmes for walking and cycling.

[B20] Deci EL, Ryan RM (1985). Intrinsic motivation and self-determination in human behavior.

[B21] Deci EL, Ryan RM (2000). The 'what' and 'why' of goal pursuits: Human needs and the self-determination of behavior. Psychol Inq.

[B22] Williams GC, Rodin GC, Ryan RM, Grolnick WS, Deci EL (1998). Autonomous regulation and long-term medication adherence in adult outpatients. Health Psychol.

[B23] Williams GC, Gagné M, Ryan RM, Deci EL (2002). Facilitating autonomous motivation for smoking cessation. Health Psychol.

[B24] Williams GC, McGregor HA, Sharp D, Lévesque C, Kouides RW, Ryan RM, Deci EL (2006). Testing a self-determination theory intervention for motivating tobacco cessation: supporting autonomy and competence in a clinical trial. Health Psychol.

[B25] Williams GC, Grow VM, Freedman ZR, Ryan RM, Deci EL (1996). Motivational predictors of weight loss and weight-loss maintenance. J Pers Soc Psychol.

[B26] Williams GC, McGregor HA, Zeldman A, Freedman ZR, Deci EL (2004). Testing a self-determination theory process model for promoting glycemic control through diabetes self-management. Health Psychol.

[B27] Wilson PM, Rodgers WM, Blanchard CM, Gessell J (2003). The relationship between psychological needs, self-determined motivation, exercise attitudes, and physical fitness. J Appl Soc Psychol.

[B28] Wilson PM, Rodgers WM (2004). The relationship between perceived autonomy support, exercise regulations and behavioral intentions in women. Psychol Sport Exerc.

[B29] Edmunds JK, Ntoumanis N, Duda JL (2006). A test of self-determination theory in the exercise domain. J Appl Soc Psychol.

[B30] Edmunds JK, Ntoumanis N, Duda JL (2007). Testing a self determination focussed teaching style in the exercise domain. Eur J Soc Psychol.

[B31] Edmunds JK, Ntoumanis N, Duda JL (2007). Adherence and well-being in obese patients referred to an exercise on prescription scheme: A self-determination theory perspective. Psychol Sport Exerc.

[B32] Brandon PR, Taum AKH, Young DB, Pottenger FM (2008). The development and validation of the Inquiry Science Observation Coding Sheet. Eval Program Plann.

[B33] Rouse PC, Ntoumanis N, Duda JL (2008). The Development of an Observational Assessment Tool Examining Environmental Support within Physical Activity Consultation. J Sports Sci.

[B34] Williams GC, McGregor H, Sharp D, Kouides RW, Lévesque CS, Ryan RM, Deci EL (2006). A Self-Determination Multiple Risk Intervention Trial to Improve Smokers' Health. J Gen Intern Med.

[B35] Hillsdon M, Foster C, Cavill N, Crombie H, Naidoo B (2005). The effectiveness of public health interventions for increasing physical activity among adults: a review of reviews.

[B36] Kirk A, Barnett J, Mutrie N (2007). Physical activity consultation for people with Type 2 diabetes. Evidence and guidelines. Diabet Med.

[B37] Kaplan SH, Greenfield S, Ware JE (1989). Assessing the Effects of Physician-Patient Interactions on the Outcomes of Chronic Disease. Med Care.

[B38] Mutrie N, Carney C, Blamey A, Crawford F, Aitchison T, Whitelaw A (2002). "Walk in to Work Out": a randomised controlled trial of a self help intervention to promote active commuting. J Epidemiol Community Health.

[B39] Diabetes Prevention Program Research Group (2002). Reduction in the Incidence of Type 2 Diabetes with Lifestyle Intervention or Metformin. N Engl J Med.

[B40] Michie S, Abraham C (2004). Interventions to change health behaviours: Evidence-based or evidence-inspired. Psychol Health.

[B41] Rollnick S, Butler CC, McCambridge J, Kinnersley P, Elwyn G, Resnicow K (2005). Consultations about changing behaviour. BMJ.

[B42] Salis JF, Haskell WL, Wood PD, Fortmann SP, Rogers T, Blair SN, Paffenbarger RS (1985). Physical activity assessment methodology in the Five-City Project. Am J Epidemiol.

[B43] Nelson EC, Wasson J, Kirk J, Keller A, Clark D, Stewart A, Zubkoff M (1987). Assessment of function in routine clinical practice: description of the COOP Chart method and preliminary findings. J Chronic Dis.

[B44] Jenkinson C, Mayou R, Day A, Garratt A, Juszczac E (2002). Evaluation of the Dartmouth COOP Charts in a large-scale community survey in the United Kingdom. J Public Health Med.

[B45] Zigmond A, Snaith R (1983). The Hospital Anxiety and Depression Scale. Acta Psych Scand.

[B46] Ryan R, Frederick C (1997). On energy, personality and health: Subjective vitality as a dynamic reflection of wellbeing. J Pers.

[B47] Bostic TJ, Rubio DM, Hood M (2000). A validation of the subjective vitality scale using structural equation modeling. Soc Indic Res.

[B48] Wilson PM, Rogers WT, Rodgers WM, Wild TC (2006). The Psychological Need Satisfaction in Exercise Scale. J Sport Exerc Psychol.

[B49] Markland D, Tobin V (2004). A modification of the behavioural regulation in exercise questionnaire to include an assessment of Amotivation. J Sport Exerc Psychol.

[B50] Jakicic JM, Marcus BH, Gallagher KI, Napolitano M, Lang W (2003). Effect of exercise duration and intensity on weight loss in overweight sedentary women. JAMA.

[B51] Eldridge SM, Ashby D, Feder GS, Rudnicka AR, Ukoumunne OC (2004). Lessons for cluster randomization trials in the twenty-first century: a systematic review of trials in primary care. Clin Trials.

